# *Himasthla* spp. (Trematoda) in the edible cockle *Cerastoderma edule*: review, long-term monitoring and new molecular insights

**DOI:** 10.1017/S0031182022000373

**Published:** 2022-06

**Authors:** Anaïs Richard, Olivier Maire, Guillemine Daffe, Luísa Magalhães, Xavier de Montaudouin

**Affiliations:** 1 UMR 5805, EPOC UMR, OASU, Université de Bordeaux, F33120 Arcachon, France; 2 Université de Bordeaux, CNRS, Observatoire Aquitain des Sciences de l'Univers, UMS 2567 POREA, F-33615 Pessac, France; 3 CESAM – Centre for Environmental and Marine Studies, Departamento de Biologia, Universidade de Aveiro, 3810-193 Aveiro, Portugal

**Keywords:** Cockles, *Himasthla*, metacercariae, phenology, trematode parasites

## Abstract

Trematodes are the main macroparasites in coastal waters. The most abundant and widespread form of these parasites is metacercaria. Their impact on their host fitness is considered relatively low but metacercarial larvae of some species can have deleterious effects on individuals and/or populations. This review focused on the cockle *Cerastoderma edule* and four species of the genus *Himasthla*; a common host–parasite system in marine coastal environments. Our aims were (1) to review literature concerning *Himasthla continua*, *Himasthla elongata*, *Himasthla interrupta* and *Himasthla quissetensis* in cockles; (2) to provide molecular signatures of these parasites and (3) to analyse infection patterns using a 20-year monthly database of cockle monitoring from Banc d'Arguin (France). Due to identification uncertainties, the analysis of the database was restricted to *H. interrupta* and *H. quissetensis*, and it was revealed that these parasites infect cockles of the same size range. The intensity of parasites increased with cockle size/age. During the colder months, the mean parasite intensity of a cockle cohort decreased, while infection occurred in the warmest season. No inter-specific competition between trematode parasites was detected. Furthermore, even if the intensity of *H. interrupta* or *H. quissetensis* infection fluctuated in different years, this did not modify the trematode community structure in the cockles. The intensity of infection of both species was also positively correlated with trematode species richness and metacercarial abundance. This study highlighted the possible detrimental role of *Himasthla* spp. in cockle population dynamics. It also revealed the risks of misidentification, which should be resolved by further molecular approaches.

## Graphical abstract

**Figure:**
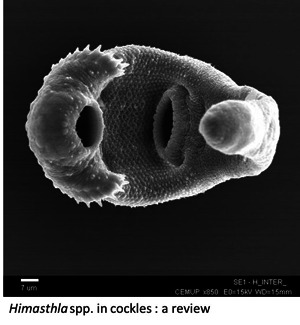

## Introduction

In coastal ecosystems, trematodes are the most abundant and common metazoan parasites (Lauckner, 1983; Sousa, 1991; Mouritsen and Poulin, 2002). These macroparasites are exclusively endoparasites and have a complex and heteroxenous life cycle, generally involving three hosts and exhibiting alternation between asexual multiplication and sexual reproduction phases (Esch, 2002; Bartoli and Gibson, 2007). The adult stage of these parasites reproduces sexually in the final host, which is a vertebrate (generally a fish or a shorebird). Eggs are released into the environment (through final host feces) and either evolve into miracidium, a free-living stage, to infect the first intermediate host (usually a mollusc), or hatches in miracidium after they have been ingested by the first intermediate host. Each larva develops into a sac-like sporocyst or a redia, depending on the trematode species, which will asexually produce cercariae, a second free-living stage. These cercariae emerge from the first intermediate host and swim actively to penetrate the second intermediate host (a vertebrate or an invertebrate) and settle as a metacercaria, a latent stage. When the second host is predated by the final host, metacercaria transforms into the adult stage, achieving the life cycle.

Molluscs are the common first and second intermediate hosts of trematode parasites and almost all known bivalves are parasitized, with predominant infection by metacercariae compared to infection by sporocysts (Lauckner, 1983; Sousa, 1991; Galaktionov and Dobrovolskij, 2003). More particularly, the edible cockle *Cerastoderma edule* (Linnaeus, 1758) is one of the most widespread and abundant bivalves in soft bottom shallow coastal ecosystems along the northeast Atlantic (Malham *et al*., 2012). They are suitable hosts for harbouring one of the highest diversities of trematode species (Krakau *et al*., 2006; Thieltges *et al*., 2006), with 16 known species (de Montaudouin *et al*., 2021). Living buried a few centimetres into the sediment, this bivalve is a key species in coastal ecosystem functioning (Carss *et al*., 2020). In particular, due to their bioturbation (i.e. biomixing of the sediment) and biodeposition activities, they modulate the physical properties and biogeochemical dynamics of the sediment (Ciutat *et al*., 2007; Eriksson *et al*., 2017; Dairain *et al*., 2020*b*), and they have an important role in connection between trophic levels (Rakotomalala *et al*., 2015). Finally, through their filtration activity, cockles can regulate phytoplankton biomass and turbidity (Cloern, 1982; Newell, 2004). The effect of trematodes is not restricted to cockle activity, but may significantly alter their fitness, with a subsequent impact at the population scale. Indeed, parasites can significantly contribute to *C. edule* mortality and population decline (Burdon *et al*., 2014). The influence of trematode parasites on cockle survival is highly dependent on species, infection intensity (i.e. number of parasites per infected cockle) or abundance (i.e. number of parasites per cockle, infected or not), prevalence (i.e. percentage of infected cockles) and parasitic stage (Lauckner, 1983). When cockles are the first intermediate hosts, the effects are particularly deleterious. For example, *Bucephalus minimus* sporocysts invade most of the tissues, including the gonads, digestive gland and gills (Dubois *et al*., 2009). This invasion leads to castration, starvation, reduction of the cockle growth rate and condition index, as well as modulation of their impact on sediment erodibility (de Montaudouin *et al*., 2009, 2021; Magalhães *et al*., 2015; Dairain *et al*., 2020*a*). In addition, *Gymnophallus choledochus*, also using *C. edule* as first (and second) intermediate host, occupies the entire mantle cavity, causing gonad structure loss and mass mortality (Thieltges, 2006*b*; Magalhães *et al*., 2020*a*). Nevertheless, prevalence is usually low and the effect on the cockle population scale is considered moderate (Thieltges *et al*., 2008; de Montaudouin *et al*., 2009, 2021). Metacercariae have a more limited impact since they do not multiply in their second intermediate host tissues. For instance, *Renicola roscovitus* is one of the dominant metacercariae encysting in cockle palps (Krakau *et al*., 2006; Lassalle *et al*., 2007). Its impact on cockles has been reported as moderate, with a slight reduction in oxygen consumption, no impact on the condition index, low antioxidant defence activation and an intermediate level of cellular damage (Magalhães *et al*., 2020*b*). However, other species of trematodes infecting cockles as metacercariae can have more deleterious impacts on their host population, especially when the prevalence and intensity become high. For example, *Gymnophallus minutus* (de Montaudouin *et al*., 2000; Thieltges and Reise, 2006*a*; Gam *et al*., 2008; Fermer *et al*., 2010) causes pathology in cockles, modifies their behaviour (emerging at the sediment–water interface) and provokes significant mortality (Bowers *et al*., 1996; Gam *et al*., 2009*b*; Fermer *et al*., 2011*a*).

The *Himasthla* genus occurs along the northeastern Atlantic coasts (James, 1968; Blakeslee and Byers, 2008, Galaktionov *et al*., 2021), and four species constitute the subject of this review (i.e. *Himasthla continua*, *Himasthla elongata*, *Himasthla interrupta* and *Himasthla quissetensis*). This is one of the most prevalent, abundant and widespread trematode genera infecting cockles as the second intermediate host (Thieltges and Reise, 2006*a*; Gam *et al*., 2008; de Montaudouin *et al*., 2021). The aims of this review were: (1) to compile literature concerning these parasites in cockles and to summarize the main findings; (2) to provide a molecular signature with the potential to accompany stereomicroscope morphological identification and (3) to analyse a 20-year long-term database concerning a cockle population and its associated trematode species in Banc d'Arguin, France, in order to describe the infection patterns of cockles by *Himasthla* spp. In the latter case, the tested hypotheses were: (1) infection increases with age and with seasonal modulation; (2) infection success may be limited by cockle abundance (dilution effects) and (3) *Himasthla* species occupy different ecological niches (i.e. different organs in the cockle) and do not compete inside their individual host.

## Materials and methods

### Literature review

The references gathered in this review were found in Scopus using relevant terms such as ‘*Cerastoderma* (or *Cardium*) *edule*’ and ‘*Himasthla*’, published before March 2021. The list of articles was restricted to those studies that clearly identified the occurrence of *H. continua*, *H. elongata*, *H. interrupta* and *H. quissetensis* in *C. edule*. A reference list of relevant papers was provided and their main findings were summarized. Thus, a total of 46 publications was examined.

### Long-term monitoring

#### Sampling and trematode identification

From November 1997 to October 2018, cockle monitoring was performed in Banc d'Arguin (44°40′N; 1°10′W), a National Nature Reserve in France. The sampled station is an intertidal semi-sheltered sandflat. The sediment is composed of medium sands (grain-size median = 330 *μ*m) (de Montaudouin and Lanceleur, 2011), the temperature of water fluctuates seasonally between 9.5 and 21.5°C and the salinity is constant (34–35). The tide is semidiurnal (Gassiat, 1989). Cockles were collected monthly by sampling six 0.25 m^2^ quadrates sieved with a 1 mm mesh. Cockle shell length was measured to the nearest millimetre with a digital calliper. Cohorts were identified by the analysis of length frequency histograms (Bhattacharya, 1967). Ten cockles per cohort were dissected and squeezed between two glass slides for trematode observation under a stereomicroscope. All trematodes were identified to the species level using morphological criteria (de Montaudouin *et al*., 2009, 2021). However, the different species of the *Himasthla* genus remain difficult to distinguish using morphological analysis and light microscopy. Therefore, several metacercariae were punctually dissected in different cockle tissues, identified morphologically under the microscope (size, number of spines) and then molecularly characterized (see the molecular biology section). Four species were identified in Banc d'Arguin: *H. continua*, *H. elongata*, *H. interrupta* and *H. quissetensis*. This long-term monthly survey was not based on the dissection of all metacercariae, so that our present analysis was restricted to *H. interrupta* and *H. quissetensis*. Indeed, we noticed frequent mistakes concerning stereomicroscope identification between the two other species: *H. continua* and *H. elongata*.

### Data analysis

**Fig. 1.**
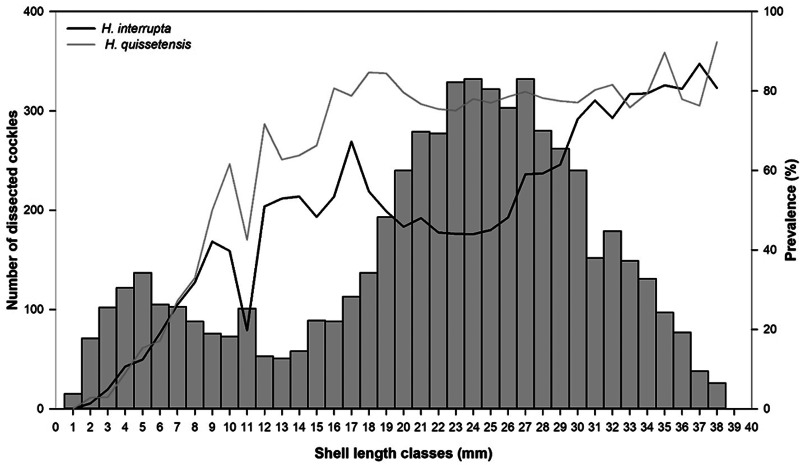
Prevalence of *Himasthla interrupta* (black line) and *Himasthla quissetensis* (grey line) by shell length class and number of dissected cockles (bars).

During the 20 years, 5820 cockles were analysed (Fig. 1), with shell lengths ranging between 2 and 38 mm. A Spearman test was conducted to investigate the relationship between the prevalence (i.e. the percentage of infected cockles) and the cockle shell length for both *Himasthla* species. Then, the seasonality of the intensity of infection (i.e. number of metacercariae per infected cockle) was studied. Firstly, cockles’ shell lengths were transformed into relative age using a local Von Bertalanffy growth function (Gam *et al*., 2009*b*):
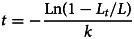
where *t* is the relative cockle age (years), *L_t_* is the cockle length at age *t* (mm), *k* = 1.5 year^−1^ and *L*_∞_ = 38.3 mm. Absolute age was deduced from a probable recruitment date in May (de Montaudouin *et al*., 2021). Then, for each *Himasthla* species, parasite intensity was compared between months (i.e. between cockle age), using a Wilcoxon non-parametric test, in order to detect significant infection (i.e. increase of the parasite intensity) or parasite-dependent mortality (i.e. decrease of the parasite intensity) processes.

For the following tests, all 1-year-old cockles (corresponding to 15–25 mm) were pooled. This cockle range was selected in order to exclude younger cockles, which are always poorly infected (whatever the environmental conditions are) and older cockles, which do not occur every year. For each *Himasthla* species, the associated trematode community was compared at the cockle specimen scale with and without this parasite species, through relative abundance (*χ*^2^ test), species richness and metacercariae abundance (Wilcoxon test). The effect of cockle density on *Himasthla* infection was tested with a Spearman correlation test. Trematodes using cockles as first intermediate hosts were not considered (*Bucephalus minutus*, *G. choledochus*, *Monorchis parvus*), as they have recently been studied in detail (Magalhães *et al*., 2015, 2020*a*). All statistical analyses were performed using the open-source program R (v3.6.1) in R studio (v1.3.1056) (www.R-project.org, accessed on 1 August 2020).

### Molecular identification: DNA isolation, amplification and sequencing

The cockles were dissected to extract metacercariae from the four *Himasthla* species that can occur in cockles. Prior to molecular biology analysis, identification was performed based on morphology (metacercariae diameter and number of oral spines) and tissue location (mantle, foot, digestive gland). Metacercariae were assigned to *H. quissetensis* when 31 oral spines were present (Stunkard, 1938). Metacercariae were confidently assigned to *H. interrupta* when they presented 29 oral spines with a diameter <140 *μ*m, and occurred in the mantle margin (de Montaudouin *et al*., 2009). A mismatch between *H. continua* and *H. elongata* was possible when metacercariae had 29 spines with a diameter >150 *μ*m and occurred in the foot. Periwinkles (*Littorina littorea*) were also collected as the first intermediate host of *H. elongata*. These periwinkles were disposed in small dishes at ambient temperature in order to stimulate cercariae emission (Wegeberg *et al*., 1999). Then, cercariae were sampled with a micropipette for molecular analysis and subsequent comparison with large 29-spine metacercariae found in the cockle foot.

Metacercariae and cercariae were sampled under a stereomicroscope for DNA analysis. For all species, three replicates (i.e. metacercariae) were collected. They were placed in microtubes and immediately frozen at −20°C. DNA extraction was performed using the QIAamp DNA micro kit (QIAGEN, Hilden, Germany), following the protocol supplied by the manufacturer. Using primers Bb18S and Bb18AS for small subunit ribosomal RNA gene (18S) (de Montaudouin *et al*., 2014), BbITS and BbITAS for internal transcribed spacer 1 (ITS1) (de Montaudouin *et al*., 2014) and TremCOIS2 and TremCOIAS2 for cytochrome c oxidase subunit I (COI) (Magalhães *et al*., 2020*a*), with sequences given in Table 1, about 530 bp of 18S, 600 bp for ITS1 and 300 bp of COI genes were amplified. The polymerase chain reaction (PCR) was performed with Gotaq G2 Flexi DNA polymerase (PROMEGA, Madison, Wisconsin, USA), with 50 *μ*L mixtures containing: 10 *μ*L of 5× Colorless GoTaq^®^ reaction buffer (final concentration of 1×), 1.5 *μ*L of MgCl_2_ solution (final concentration of 1.5 mm), 1 *μ*L of PCR nucleotide mix (final concentration of 0.2 mm each dNTP), 0.5 *μ*L of each primer (final concentration of 1 *μ*m), 0.2 *μ*L of GoTaq^®^ G2 Flexi DNA polymerase (5 U *μ*L^−1^), 1 *μ*L template DNA and 33.8 *μ*L of nuclease-free water. The temperature profile was 94°C/10 min–(94°C/60 s–59°C/30 s–72°C/90 s) × 40 cycles–72°C/10 min–4°C for 18S and ITS1, and 95°C/10 min–(95°C/60 s–43°C/30 s–72°C/60 s) × 40 cycles–72°C/10 min–4°C for COI. The amplified PCR products were analysed by electrophoresis in a 1% p/v agarose gel stained with ethidium bromide. They were then sent to Eurofins Company for complete double-strain sequencing, using the same set of primers as used for the PCR. Overlapping sequence (forward and reverse) fragments were merged into consensus sequences and aligned using Clustal Omega. For COI, the sequences were translated into amino acid alignments, and checked for stop codons to avoid pseudogenes. All sequences obtained in this study were deposited in GenBank (Table 2).

**Table 1.** Nucleotide sequences of specific primer pairs

Primer name	Used for	Primer sequence	Reference
Bb18S	PCR and sequencing	5′-ACTGGAGGGCAAGTCTGGTGC-3′	de Montaudouin *et al*. (2014)
Bb18AS	PCR and sequencing	5′-CAGCTTTGCAACCATACTTCCC-3′	de Montaudouin *et al*. (2014)
BbITS	PCR and sequencing	5′-GACCGAACTTGATCATTTAGAGG-3′	de Montaudouin *et al*. (2014)
BbITAS	PCR and sequencing	5′-CTTAAGTTCAGCGGGTAATCACG-3′	de Montaudouin *et al*. (2014)
TremCOIS2	PCR and sequencing	5′-TGTTYTTTAGKTCTGTKAC-3′	Magalhães *et al*. (2020*a*)
TremCOIAS2	PCR and sequencing	5′-AATGCATMGGRAAAAAACA-3′	Magalhães *et al*. (2020*a*)

**Table 2.** Accession numbers when DNA sequences were deposited in GenBank, for each gene (18S, ITS1 and COI) and the four *Himasthla* species

Species	18S	ITS1	COI
*Himasthla continua*	–	–	–
*Himasthla elongata*	MN879359	MN876024	MT002921
*Himasthla interrupta*	MN879360	–	MT002922
*Himasthla quissetensis*	MN879357	MN876026	MT002919

## Results

### Literature review

#### Description and life cycle

*Himasthla continua* (Loos-Frank, 1967), *H. elongata* (Mehlis, 1831) Dietz, 1909, *H. interrupta* (Loos-Frank, 1967) and *H. quissetensis* (Miller & Northup, 1926) Stunkard, 1938, belong to the Platyhelminthes phylum, Trematoda class, Digenea subclass and Himasthlidae family (Table 3). *Himasthla continua*, *H. elongata* and *H. interrupta* are considered to be native parasites of *C. edule*, whereas *H. quissetensis* could have been introduced to Europe from North America (de Montaudouin *et al*., 2005; Longshaw and Malham, 2013), and was not reported before 1990 on the eastern Atlantic coastline (Russell-Pinto, 1993). *Himasthla* species can be differentiated by morphometric measures, number of spines and to a lesser extent by their location in cockle organs (Russell-Pinto *et al*., 2006; de Montaudouin *et al*., 2009). *Himasthla quissetensis* is the only one of these four species with 31 oral spines. *Himasthla interrupta* displays the smallest cyst diameter (80–140 *μ*m) and is mainly located in the cockle mantle margin (in the anterior edge, at the opposite side of siphons). In contrast, *H. elongata* displays the largest cysts (210–270 *μ*m). The size of *H. continua* metacercariae ranges between 150 and 210 *μ*m.

**Table 3.** Characteristics of the four studied *Himasthla* species in terms of target organs, number of oral spines, metacercariae mean diameter and different host species within their life cycle

Trematode species (Echinostomatoidea, Himasthlidae)	Synonyms	Organs affected	Morphological identification	First intermediate host	Second intermediate host	Final host
*Himasthla elongata* (Mehlis, 1831; Dietz, 1909)	*Himasthla secunda*	Foot	29 oral spines, diameter: 210–270 *μ*m	*Littorina littorea*	*Cerastoderma edule* and other bivalves	Seagull
	*Echinostomum secundum*					
	*Distoma elongata*					
*Himasthla interrupta* Loos-Frank (1967)	–	Mantle	29 oral spines, diameter: 80–140 *μ*m	*Peringia ulvae*	*C. edule* and other bivalves	Sandpiper
*Himasthla continua* Loos-Frank (1967)	–	Foot, sometimes mantle	29 oral spines, diameter: 150–210 *μ*m	*P. ulvae*	*C. edule* and other bivalves	Seagull
*Himasthla quissetensis* (Miller and Northup, 1926; Stunkard, 1938)	–	Foot, sometimes mantle	31 oral spines, diameter: 150–210 *μ*m Tritia neritea	*Tritia reticulata*	*C. edule* and other bivalves	Seagull

#### Geographic distribution, abundance and prevalence

*Himasthla* spp. metacercariae infect *C. edule* from Norway to North Africa, with different successes according to the species (Table 4). *Himasthla continua* and *H. interrupta* are ubiquitous species, present from Denmark to Morocco. They use the same first intermediate host, *Peringia ulvae* (Bordalo *et al*., 2011), which is also widely distributed along the east Atlantic shoreline and could explain that they follow similar distribution patterns. However, the intensity of infection and the prevalence are generally higher for *H. interrupta* than those for *H. continua* (de Montaudouin *et al*., 2009). This can be attributed to the fact that *H. continua* cercariae have more difficulties penetrating through the cockle inhalant siphon due to their larger dimension (Wegeberg *et al*., 1999). *Himasthla elongata* is absent South to Portugal, and the highest abundance occurs in the northern European countries (e.g. Norway) (de Montaudouin *et al*., 2009). This range could be related to the distribution of the first intermediate host, the periwinkle *L. littorea* which is present from Portugal to Norway and Russia (Johannesson, 1988). In contrast, *H. quissetensis* is mainly reported in the southern part of the *C. edule* geographical distribution, with the highest rate of infection in France, Portugal and Morocco. In this case, the first intermediate host is *Tritia reticulata*, which is widespread from the north to south of Europe (Russell-Pinto *et al*., 2006). This lack of direct relationship between host and parasite distribution shows that the abundance of metacercariae also depends on other factors, such as cockle density, size, age and fitness, as well as the ambient benthic community (Gam *et al*., 2009*b*; de Montaudouin and Lanceleur, 2011; Magalhães *et al*., 2017; Welsh *et al*., 2019; Correia *et al*., 2020*a*). Moreover, *H. quissetensis* has also been recorded in different Mediterranean lagoons, infecting a close-related cockle, *C*erastoderma *glaucum* (Prévot, 1974; Bartoli and Gibson, 2007).

**Table 4.** Review of the literature regarding *H. continua*, *H. elongata*, *H. interrupta* and *H. quissetensis* metacercariae infection in *C. edule*

Reference	Location	Trematode species	*C. edule* size	Abundance (Ab) or intensity (I)	Prevalence (%)	Main findings
Lebour (1911)	British waters	*H. elongate* (*E. secundum*)	–	Occurrence	10	Description
Kesting *et al*. (1996)	Baltic Sea, Germany	*H. interrupta*		Occurrence	0–5	Adaptation of trematode parasites to low salinity environment
de Montaudouin *et al*. (1998)	Wadden Sea, Denmark	*H. elongata*	6–12 mm	–	–	Infection intensity of *H. elongata* increased with cockle density; passive infection of cockles as second intermediate host; the dispersion of *H. elongata* cercariae was at least hundred metres
Jensen *et al*. (1999)	Arcachon, France	*H.* spp. (mainly (98%) *H. continua*)	1–6 mm	–	–	Cockles size selection (threshold of 2/3 mm); no increase of cockle mortality by *H.* spp. metacercariae but slight prolongation of burrowing time
Wegeberg and Jensen (1999)	Wadden Sea, Germany	*H. elongata*	9.5–11 mm	Occurrence	–	*H. elongata* reduced the burrow ability and the survival of cockles under hypoxic condition
		*H. continua*		Occurrence	–	
		*H. interrupta*		Occurrence	–	
Wegeberg *et al*. (1999)	Wadden Sea, Germany	*H. continua*	–	–	–	*H. interrupta* has high infectivity in cockles measuring 4 mm while *H. continua* and *H. elongata* have low infection efficiencies in cockles less than 6 mm
		*H. interrupta*		–	–	
		*H. elongata*		–	–	
de Montaudouin *et al*. (2000)	Arcachon Bay, France	*H. continua*	Infection started at 12 mm	Ab: 0–75	47.9	Occurrence; *H. continua* and *H. interrupta* dominant in *C. edule*; parasite species richness increased with cockle shell length; infection of cockles increases in summer
		*H. interrupta*	Infection started at 7 mm	Ab: 0–160	37.5	
		*H. elongata*	–	Occurrence	0.7	
			
Desclaux *et al*. (2002)	Arcachon, France	*H. quissetensis*	<33 mm	Ab: 0–220	Up to 100	*H. quissetensis* abundance was slightly higher in surface cockles at Banc d'Arguin than buried cockles; 2–8% of adult cockles emerged due to favourization process by *H. quissetensis*; favourization mechanism induced by trematodes did not explain the magnitude of cockles mortality; similar parasites community between buried and surfaced cockles
		*H. interrupta*		Occurrence	Up to 30	
		*H. elongata*		Occurrence	<5	
Blanchet *et al*. (2003)	Arcachon, France	*H.* spp.	24 mm	Ab: up to 566 (mean: 36)	100	*H.* spp. corresponded to *H. interrupta* and *H. quissetensis*; only bacteria could trigger the cockle emergence and affected their survival
			27 mm	Ab: up to 560 (mean: 56)		
Wegeberg and Jensen (2003)	Wadden Sea, Denmark	*H. interrupta*	3–8 mm	Ab: 0.15	15	Under normal environmental condition, moderate infection of *H. interrupta* metacercariae did not significantly affect cockle mortality or their shell growth
Desclaux *et al*. (2004)	Arcachon, France	*H. quissetensis*	<35 mm	Ab: 0–120	–	*H. quissetensis* infection occurred during the warmest period and significantly contributed to cockle mortality (up to 46%); only cockles >8 mm were infected; cockle mortality due to parasites depended on cockle growth and environmental factors
		*H. interrupta*		Occurrence	–	
		*H. elongata*		Occurrence	–	
de Montaudouin *et al*. (2005)	Arcachon, France	*H. continua*	2–14 mm	Ab: 0	–	Infection pattern of *H. quissetensis* was similar to those of *H. elongata* and *H. continua*; *H. quissetensis* has high infectivity in cockles measuring 6–14 mm
		*H. interrupta*		Ab: 0–2.7	–	
		*H. elongata*		Ab: 0–0.5	–	
		*H. quissetensis*		Ab: 0–3.2	–	
Baudrimont and de Montaudouin (2006)	Arcachon, France	*H. quissetensis*	25.2 ± 0.4 mm	Ab: 12.5 ± 2.2	–	Alteration of the metallothioneins protective effect on parasitized cockles after cadmium exposure at the whole organism level
			27.9 ± 0.2 mm	Ab: 1.5 ± 0.2		
Baudrimont *et al*. (2006)	Arcachon, France	*H. quissetensis*	23–31 mm	Ab: 10–90	–	Parasite infection in cockles altered the protective effect (metallothioneins synthesis) in case of metal contamination
Krakau *et al*. (2006)	Wadden Sea, Germany	*H. continua*	26–47 mm	I: 5.6 ± 0.9 to 26.6 ± 7.5	20–61.5	Similar trematode parasite species were found in native and introduced host species, but intensity of infection was higher in native host species
		*H. interrupta*		I: 11.0 ± 1.6 to 98.0 ± 11.3	5–100	
		*H. elongata*		I: 7.5 ± 1.7 to 48.8 ± 10.4	37.5–94.9	
Russell-Pinto *et al*. (2006)	Ria de Aveiro, Portugal	*H. interrupta*	–	Occurrence	4.23	Species description, identification and distribution
		*H. elongata*		Occurrence	26.88	
		*H. quissetensis*		Occurrence	78.54	
Thieltges (2006*b*)	Wadden Sea, Germany	*H. continua*	(2/3 years)	Ab: 148.4 ± 111.1 to 164.2 ± 84.4	–	Negligible role of metacercariae in cockle mortality
		*H. interrupta*		metacercariae (*H.* 3 spp.) in buried cockles		
		*H. elongata*			–	
					–	
Thieltges (2006*a*)	Wadden Sea, Germany	*H. continua*	Cockles with 2 winter rings	0 to ~ 2100 metacercariae per g flesh dry weight	–	Density of the first intermediate host was the dominant factor of metacercarial transmission; tidal level was a minor factor in trematode transmission
		*H. interrupta*		0 to ~ 3200 metacercariae per g flesh dry weight		
		*H. elongata*		0 to ~ 500 metacercariae per g flesh dry weight		
Thieltges and Reise (2006*a*)	Wadden Sea, Germany	*H. continua*	20–30 mm (adult)	I: 9.0 ± 4.7 to 9.8 ± 5.8	95.8 ± 4.2 to 92.0 ± 10.7	Species richness and intensity of infection increased with cockles age; trematodes community did not vary between years, especially in adults
		*H. interrupta*		I: 126.8 ± 147.2 to 149.3 ± 225.4	100 ± 0	
		*H. elongata*		I: 16.3 ± 26.0 to 17.5 ± 22.1	70.5 ± 44.5 to 75.0 ± 46.3	
		*H. continua*	6–14 mm (juvenile)	I: 4.1 ± 3.7 to 10.5 ± 4.3	69.5 ± 23.4 to 89.5 ± 18.4	
		*H. interrupta*		I: 3.8 ± 1.9 to 21.9 ± 30.0	76.0 ± 28.9 to 95 ± 6	
		*H. elongata*		I: 2.5 ± 1.1 to 7.6 ± 3.9	50.0 ± 33.4 to 96.5 ± 4.1	
Thieltges *et al*. (2006)	Wadden Sea, Germany	*H. continua*	21–47 mm	I: 15.8 ± 25.1	72.3 ± 34.7	Occurrence, *H. quissetensis* was absent probably due to the lack of its first intermediate host
		*H. interrupta*		I: 43.7 ± 20.8	84.2 ± 38.8	
		*H. elongata*		I: 20.5 ± 24.7	85.7 ± 21.4	
Desclaux-Marchand *et al*. (2007)	Arcachon, France	*H. elongata*	>20 mm	–	–	Trematode parasite increased metallothioneins concentration (metal-binding proteins) in gills
Javanshir *et al*. (2007)	Arcachon, France	*H.* spp.	>20 mm	I: 6.87–11.8	–	Intensity of infestation by *H.* spp. metacercariae was higher in cockles initially infected than in cockles without metacercariae; number of metacercariae increased with cockle shell length and density (higher filtration rates); infestation slightly decreased cockle growth rates
Lassalle *et al*. (2007)	French Atlantic coast	*H. continua*	24 ± 5 mm	Occurrence	–	No relationship between abundance of metacercariae and latitude; *H. quissetensis* and *H. continua* were ubiquitous species while *H. elongata* characterized northern stations and *H. interrupta* southern stations
		*H. interrupta*		Occurrence	–	
		*H. elongata*		Occurrence	–	
		*H. quissetensis*		Occurrence	–	
Thieltges and Reise (2006*b*)	Wadden Sea, Germany	*H. continua*	18–30 mm	I: 0.1–88.2	From 7 to 100	Prevalence and intensity of metacercariae infection on cockles between sites mainly depended on the density of the first intermediate host; predictors were more complex within sites; cockle size and density were respectively positively and negatively correlated with infection level; time spent in residual water increased infection level in cockles; no interspecific interaction between trematode parasites in hosts
		*H. interrupta*		I: 0.7–136.3	From 47 to 100	
		*H. elongata*		I: 0–72.5	From 0 to 100	
Gam *et al*. (2008)	Merja Zerga, Morocco	*H. interrupta*	17 ± 1 to 30 ± 2 mm	Ab: 0–0.5	Low	Different sub-communities of metacercariae trematode parasites between subtidal and intertidal environment (cockle density, first host)
		*H. quissetensis*		Ab: 0.1–2.3; I: 1–12	10–55	
Thieltges (2008)	Wadden Sea, Germany	*H. continua*	18–42 mm	Occurrence	<5	Temporal exposure was the main factor explaining the level of infection in cockles older than 1-year-old than host size
		*H. interrupta*		Occurrence	<5	
		*H. elongata*		Ab: 20–316	–	
de Montaudouin *et al*. (2009)	North-eastern Atlantic coast	*H. continua*	–	Ab: 0–100 (Norway–Morocco)	Close to 100%	Identification key; review of the distribution of parasites along north-eastern Atlantic coast
		*H. interrupta*	–	Ab: 0–1000 (Norway–North of France)	Close to 100%	
				0–100 (Spain–Morocco)		
		*H. elongata*	–	Ab: 0–1000 (Norway–South of France)	Close to 100%	
				0–10 (South of France–Morocco)		
		*H. quissetensis*	–	Ab: 0–1000 (North of France–Morocco)	Close to 100%	
Gam *et al*. (2009*b*)	Arcachon, France	*H. interrupta*	<40 mm	Ab: 41	7.0	Trematode metacercariae did not affect cockle production at both sites but increased cockle mortality (up to 20%); metacercariae abundance threshold where cockles were impacted was lower at Merja Zerga than Arcachon probably due to interaction with environmental factors
		*H. quissetensis*		Ab: 4.1	0.7	
	Merja Zerga, Morocco	*H. interrupta*	<30 mm	Ab: 0.2	0.0	
		*H. quissetensis*		Ab: 1.4	0.2	
Gam *et al*. (2009*a*)	Merja Zerga, Morocco	*H. interrupta*	13–24 mm	I: <4	16.7–34.4	Species richness of parasites in cockles increased with time; non-significant effect of substrate on parasite community structure (small spatial scale)
		*H. quissetensis*		I: <4	5.6–13.3	
de Montaudouin *et al*. (2010)	French Atlantic coast	*H. interrupta*	31–35 mm	Ab: 0–14	–	Cockles exposed to low stress but unfavourable environment for development
		*H. elongata*		Ab: 0–38	–	
Paul-Pont *et al*. (2010)	Arcachon, France	*H. elongata*	31.8 ± 0.3 mm	–	–	*H. elongata* metacercariae induced strong immune response and modified gene expression inducting energetic losses and oxidative stress in cockles; trematode infection limited pollutant accumulation
de Montaudouin and Lanceleur (2011)	Arcachon, France	*H. continua*	17–23 mm	Ab: 1.47	–	Parasite diagnosis was representative of cockles distributed in a radius of 20 m; infection heterogeneity at a different scale was explained by the presence of the first intermediate host, mobility of hosts and environmental parameters impacting cercariae transmission
		*H. interrupta*		Ab: 5.34	–	
		*H. elongata*		Ab: 0.11	–	
		*H. quissetensis*		Ab: 10.57	–	
Fermer *et al*. (2011*b*)	South coast of Ireland	*H. continua*	24.3 ± 3.6 to 38.1 ± 4.4 mm	I: 4 ± 7	10	Parasite trematode community was similar to that found in northern Europe; *H. quissetensis* was found in southern Europe and northern Africa but not in northern Europe probably due to the absence of its first intermediate host
		*H. interrupta*		I: 1 ± 1	6	
		*H. elongata*		I: 39 ± 56	40	
de Montaudouin *et al*. (2012*b*)	Arcachon, France	*H. continua*	<35 mm	Ab: 0–45	–	Stability of trematodes community after 8 years; vulnerable cockles range size; spatial aggregation of parasites disappeared with cockles age (parasites accumulation); *H.* spp. abundance decreased in winter (cockle mortality)
		*H. interrupta*		Ab: 1–15	–	
		*H. elongata*		Ab: 0–23	–	
		*H. quissetensis*		Ab: 0–35	–	
de Montaudouin *et al*. (2012*a*)	Arcachon, France	*H. continua*	15–25 mm	Occurrence	–	*H. interrupta* significantly reduced by 23% the growth of cockles; moderate negative effect of trematodes on growth and condition of cockles
		*H. interrupta*		Ab: 227	100	
		*H. quissetensis*		Occurrence	–	
Binias *et al*. (2014)	Arcachon, France	*H. interrupta*	28–36 mm	Ab: 3–22	–	Patchy distribution of trematode parasite in Arcachon Bay; no clear relationship with environmental factors
		*H.* spp. (*H. continua*		Ab: 9–43	–	
		and *H. quissetensis*)				
Freitas *et al*. (2014)	Ria de Aveiro, Portugal	*H. continua*	19–30 mm	I: 1 ± 2 (mean ± s.d.)	32 ± 26	New occurrence of *H. continua*; most parasites preferred muddy sand areas with euhaline conditions
		*H. interrupta*		I: 3 ± 6	30 ± 34	
		*H. elongata*		I: 6 ± 10	48 ± 43	
de Montaudouin *et al*. (2016)	Arcachon, France	*H. quissetensis*	25.5 ± 1.3 mm	Ab: 0–130	–	Importance of temperature of water and light in the infection of cockles by *H. quissetensis*
Magalhães *et al*. (2017)	Arcachon, France	*H. continua*	13–30 mm	Occurrence	–	Negative correlation between cockle density and abundance of trematode parasites in juvenile cockles (dilution effect), with a threshold at 400 adult cockles per m^2^
		*H. interrupta*		Ab: <25	–	
		*H. elongata*		Occurrence	–	
		*H. quissetensis*		Ab: <10	–	
Magalhães *et al*. (2018*a*)	Ria de Aveiro, Portugal	*H. interrupta*	26–31 mm	Rare	0.9	Low abundance of trematode species; importance of the oceanic influence; negative impact of global change on trematode community
		*H. elongata*		Ab: <1	23.9	
		*H. quissetensis*		Rare	0.9	
Magalhães *et al*. (2018*c*)	Ria de Aveiro, Portugal	*H. elongata*	13–17 mm	Ab: 0.2 ± 0.9 to 0.8 ± 1.0	–	*H. elongata* cercariae infection success increased with acidification; modification of cockles biochemical performances with climate change (may decreased survival of cockles infected by *H. elongata*)
Magalhães *et al*. (2018*b*)	Ria de Aveiro, Portugal	*H. elongata*	14–17 mm	Ab: 0.8 ± 1.0	–	Intensity of infection was positively correlated with biochemical response (higher metabolic rate); high impact of *H. elongata* metacercariae on cockle biochemical performance; trematode infection limited pollutant accumulation
Correia *et al*. (2020*a*)	North-eastern Atlantic coast	*H. continua*	23–30 mm	Occurrence	47–100	Distribution of parasites along north-eastern Atlantic coast; temperature and coastal system were one of the most important drivers for parasite infection
		*H. interrupta*		Occurrence	39–100	
		*H. elongata*		Occurrence	10–48	
		*H. quissetensis*		Ab: <200	36–100	
Correia *et al*. (2020*b*)	Ria de Aveiro, Portugal	*H. continua*	17–20 mm	Occurrence	8.1	Importance of tidal position and current velocity on cockle infection
		*H. interrupta*		Occurrence	12.2	
		*H. elongata*		Occurrence	20.9	
		*H. quissetensis*		Rarest species	1.3	
Magalhães *et al*. (2020*b*)	Ria de Aveiro, Portugal	*H. elongata*	19–21 mm	Ab: 4.3 ± 2.0	–	Two days after metacercariae infection, cockles’ metabolism was negatively affected (reduced oxygen consumption); the activity of antioxidant enzymes was increased; energy reserve of cockles would decreased at longer term
de Montaudouin *et al*. (2021)	North-eastern Atlantic coast	*H. continua*		Ab: 0–0.7	0–38	General description, pathogenicity, diagnosis and risks
		*H. interrupta*		Ab: 0–87.9	0–100	
		*H. elongata*		Ab: 0–4.7	0–97	
		*H. quissetensis*		Ab: 0–22.1	0–100	

Abundance was the number of metacercariae per infected or uninfected cockle, intensity was the number of metacercariae per infected cockle and prevalence was the percentage of infected cockles.

#### Effects of second intermediate hosts

In most studies, the pathogenicity of *Himasthla* metacercariae is reported as low in *C. edule*, as this stage is considered energetically inert (Lauckner, 1983). Indeed, laboratory and field experiments have demonstrated that, under moderate infection and normal environmental conditions, *H. continua* and *H. interrupta* do not increase cockle mortality (Jensen *et al*., 1999; Wegeberg and Jensen, 2003). Similarly, *H. interrupta* and *H. quissetensis* do not impair *C. edule* shell growth and production (Wegeberg and Jensen, 2003; Gam *et al*., 2009*b*), and *H. elongata* has no significant effect on cockle bioturbation activity (sediment reworking and bioirrigation rates) (Richard *et al*., 2021). Nevertheless, when cercariae encyst in the cockle foot, they can induce damages such as muscle fibre destruction (Jensen *et al*., 1999) through mechanical pressure and tissue lysis related to the secretion of enzymes by the cercariae (Lauckner, 1983). In addition, when the abundance of *Himasthla* spp. metacercariae exceeds a certain threshold (the value could depend on environmental conditions), cockle survival is reduced, as exemplified for the 4 species: (1) *H. elongata* induces mechanical obstruction in the cockle foot, increasing their burrowing time and making them more vulnerable to predators (Lauckner, 1983). Infection also induces a strong cockle immune response (Paul-Pont *et al*., 2010). Moreover, it modulates cockle biochemical performance and physiology by reducing their oxygen consumption, increasing antioxidant enzyme activity and modifying their energy allocation (Magalhães *et al*., 2018*b*, 2018*c*, 2020*b*). Finally, infection can significantly reduce (around 40%) cockle survival compared to non-infected cockles after 30 h under hypoxic conditions (Wegeberg and Jensen, 1999). (2) *Himasthla quissetensis* promotes cockle emergence at the sediment surface, exposing them to other threats, like predation (Desclaux *et al*., 2002) and can contribute to up to 46% of cockle population mortality (Desclaux *et al*., 2004). (3) *Himasthla interrupta* moderately significantly reduces the cockle growth rate (de Montaudouin *et al*., 2012*b*), and a marginal but significant loss of infected cockle flesh weight and body condition was observed by Wegeberg and Jensen (2003). (4) In contrast, no effect on cockles was reported concerning *H. continua*, with the exception of cockle burrowing time increasing at the sediment surface (Jensen *et al*., 1999).

### Long-term monitoring

The dataset included cockles from 2 to 38 mm, corresponding to 0+ to 3+ year old cockles. Globally, the parasite community was dominated by *G. minutus* (mean of 62.8% of the total number of metacercariae per cockle), *H. interrupta* and *H. quissetensis* (16.2 and 5.5%, respectively). The other species were *Curtuteria arguinae*, *Diphterostomum brusinae*, *H. continua*, *H. elongata*, *Psilostomum brevicolle* and *R. roscovitus*. The following results aimed to obtain a mean *Himasthla*-host phenology calculated from our 20-year monthly monitoring.

#### Himasthla interrupta

Infection by *H. interrupta* started with 2 mm cockles, and prevalence regularly increased with shell length (*ρ* = 0.88, *P* < 0.001), to attain a median asymptotic prevalence of 80% (Fig. 1).

**Fig. 2.**
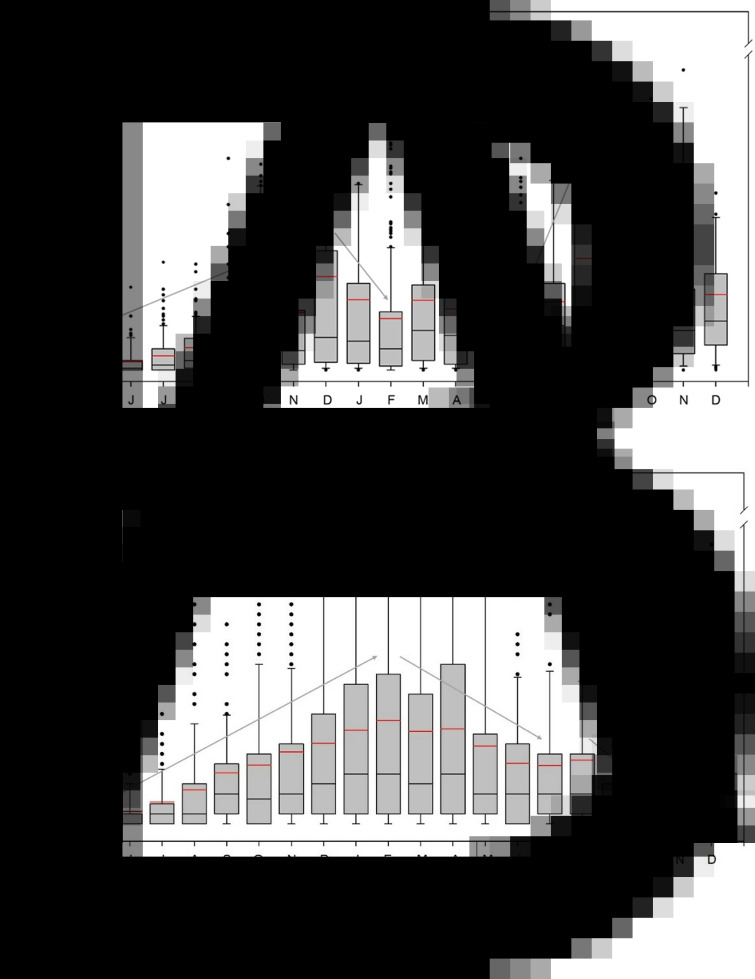
Boxplot of *H. interrupta* (A) and *H. quissetensis* (B) intensity per cockle shell length and corresponding age and seasons. Absolute age was deduced from a recruitment date in May. The box (25–75% of the data) contains a black line (median) and a red line (mean). Whiskers represent the lower and upper values in the range of ±1.5 interquartile range, with outliers as black circles. Grey arrows indicate significant variation between successive months (Wilcoxon test, *P* < 0.01). For example, in the case of *H. interrupta*, the first value that is significantly different from May 0+ intensity is in December 0+.

Mean intensity of infection increased significantly between recruitment in May [4 metacercariae per cockle, standard deviation (s.d.) = 7)] and December (50 metacercariae per cockle, s.d. = 70) (Wilcoxon test, *P* < 0.001) (Fig. 2A). Then, a significant decrease was observed until February (28 metacercariae per cockle, s.d. = 44) (*P* < 0.001), before a sharp increase during the second summer, reaching 59 metacercariae per cockle in August (s.d. = 62) (*P* < 0.001). Another decrease was observed in September (22 metacercariae per cockle, s.d. = 24) (*P* < 0.001), with a stabilization of the parasite intensity until December, which was the limit for assigning a shell length to an age, and thus identifying a cohort.

When excluding the smallest cockles (below 15 mm shell length, corresponding to 3-month old) which are rarely infected regardless of environmental conditions, and excluding the largest cockles (over 25 mm), for which age is uncertain due to slow growth, there was a weak correlation between cockle metacercariae infection and cockle density (Spearman test, *ρ* = −0.20, *P* = 0.03, not shown).

**Fig. 3.**
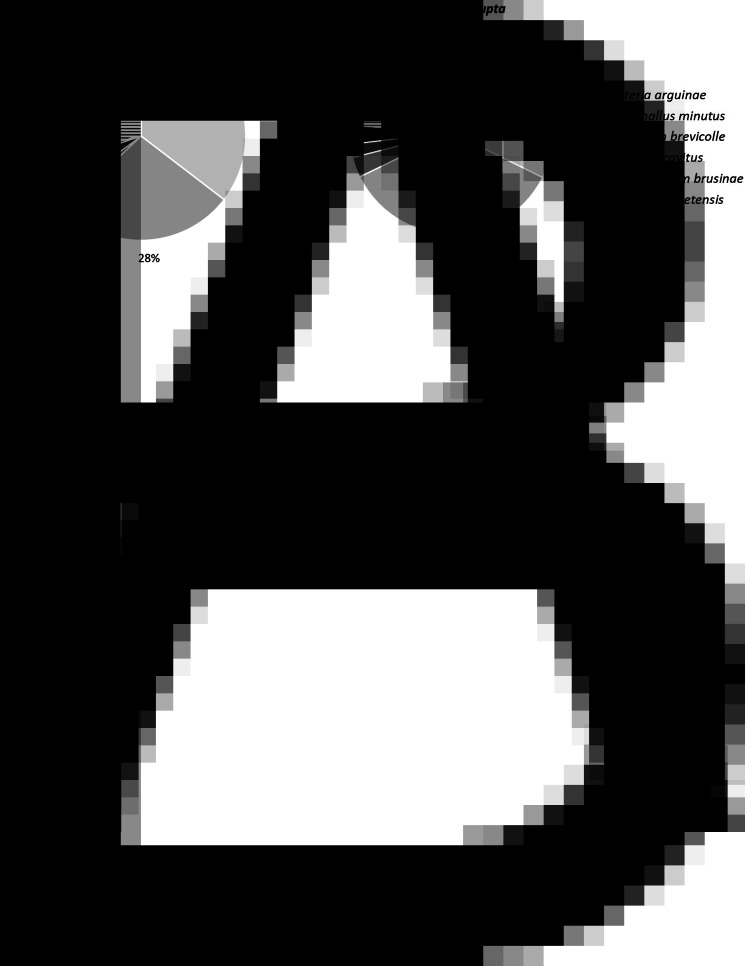
Percentage of metacercariae per species (*Curtuteria arguinae*, *Gymnophallus minutus*, *Psilostomum brevicolle*, *Renicola roscovitus*, *Diphterostomum brusinae* and *H. quissetensis*) in *Cerastoderma edule* without (A) and with (B) *H. interrupta*.

The structure of the trematode community was similar between cockles with and without *H. interrupta* (*χ*^2^ test, *P* = 0.647). *Himasthla quissetensis*, *G. minutus* and *C. arguinae* co-dominated, representing 81% of the total abundance (Fig. 3A and B). However, species richness in cockles without *H. interrupta* (2.0 species, s.d. = 1.2) was lower than in those with *H. interrupta* (3.4 species, s.d. = 1.1) (Wilcoxon test, *P* < 0.001). Similarly, the mean number of metacercariae in cockles without *H. interrupta* (28 metacercariae per cockle, s.d. = 35) was lower than those with *H. interrupta* (66 metacercariae per cockle, s.d. = 107) (Wilcoxon test, *P* < 0.001).

#### Himasthla quissetensis

Infection by *H. quissetensis* started for 2 mm cockles, and prevalence regularly increased with shell length (*ρ* = 0.78, *P* < 0.001), to reach a median asymptotic prevalence of 80% (Fig. 1). Mean intensity of infection increased significantly between recruitment in May (1 metacercariae per cockle, s.d. = 1) and February (11 metacercariae per cockle, s.d. = 14) (Wilcoxon test, *P* < 0.001) (Fig. 2B). Then, a significant decrease was observed until July (7 metacercariae per cockle, s.d. = 9) (*P* < 0.001), followed by a stagnation in summer before another decrease in October of the second year (*P* < 0.001). A late infection was observed between October and November (6 metacercariae per cockle, s.d. = 12) (*P* = 0.005). Beyond December of the second year, the correspondence between cockle shell length and cockle age determination was no longer reliable due to slower growth.

**Fig. 4.**
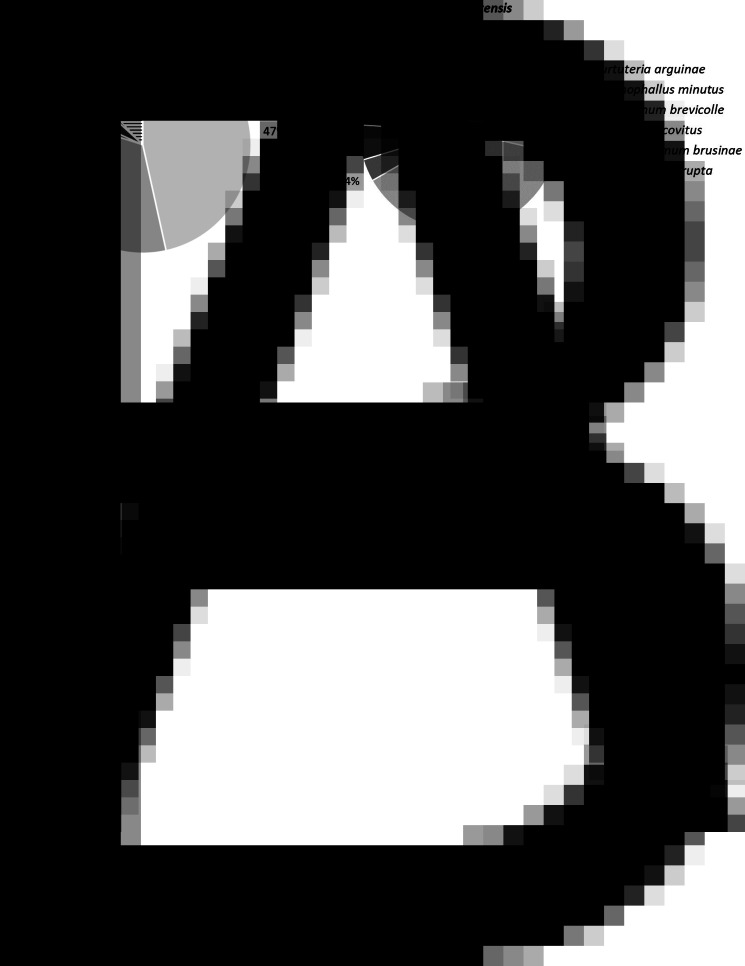
Percentage of metacercariae per species (*C. arguinae*, *G. minutus*, *P. brevicolle*, *R. roscovitus*, *D. brusinae* and *H. interrupta*) in *C. edule* without (A) and with (B) *H. quissetensis*.

For cockles with a shell length ranging between 15 and 25 mm (see section on *H. interrupta*) there was a weak correlation between cockle metacercariae infection and cockle density (Spearman test, *ρ* = −0.29, *P* < 0.001). The structure of the trematode community was similar between cockles with and cockles without *H. quissetensis* (*χ*^2^ test, *P* = 0.183). *Himasthla interrupta*, *G. minutus* and *C. arguinae* co-dominated (Fig. 4A and B). However, species richness in cockles without *H. quissetensis* (1.9 species, s.d. = 1.4) was lower than those with *H. quissetensis* (2.9 species, s.d. = 1.3) (Wilcoxon test, *P* < 0.001). Similarly, the mean number of metacercariae in cockles without *H. quissetensis* (37 metacercariae per cockle, s.d. = 78) was lower than those with *H. quissetensis* (62 metacercariae per cockle, s.d. = 101) (Wilcoxon test, *P* < 0.001).

### Molecular identification

All metacercariae from cockles were first identified under a stereomicroscope based on morphological characteristics. Then, these metacercariae were compared with molecular tools. *Himasthla continua* metacercariae are morphologically very similar to *H. elongata* (the latter are slightly larger, and they both have 29 oral spines) and both occupy the same niche (i.e. the cockle foot). This difficulty of identification explains why they were not considered in the monitoring. Additionally, no specific type sequence was determined for *H. continua* due to a large variability of gene sequences within the specimens considered as *H. continua*. Conversely, sequences of 18S, ITS and COI were obtained for *H. elongata*, and sequences of 18S and COI were confirmed from cercariae emitted by *L. littorea*. *Himasthla interrupta* metacercariae have very small and light metacercariae settled in the mantle margin, at the opposite side to the siphon. For this species, only sequences of 18S and COI were obtained, with 100% similarity between samples and 100% agreement with morphological identification. *Himasthla quissetensis* is the only species with 31 oral spines. Sequences of 18S, ITS and COI were obtained with 100% similarity between samples and 100% agreement with morphological identification. The amplified products of 18S, ITS1 and COI presented 549, 798 and 273 bp, respectively, for *H. elongata* and 516, 529 and 284 bp for *H. quissetensis*. The amplified products of 18S and COI for *H. interrupta* presented, respectively, 541 and 259 bp, and 535 and 281 bp for cercariae collected from *L. littorea*.

## Discussion

### Size- and density-dependent infection and seasonality

For both *Himasthla* species, the long-term data analysis showed that infestation started rapidly after recruitment for cockles with a 2 mm shell length. This early infection in cockles was experimentally observed for all *Himasthla* species (Jensen *et al*., 1999; Wegeberg *et al*., 1999; de Montaudouin *et al*., 2005) and is consistent with previous field studies (de Montaudouin *et al*., 2000; Desclaux *et al*., 2004). The positive relationship between parasite prevalence and cockle shell length was also documented and ascribed to the higher filtration rate and longer exposure time of older/larger individuals (André *et al*., 1993; Riisgård, 2001), resulting in a higher exposure to infective stages and thus greater parasite accumulation (de Montaudouin *et al*., 1998; Mouritsen *et al*., 2003; Thieltges and Reise, 2006*a*).

A moderate negative correlation between cockle density and intensity of *H. interrupta* and *H. quissetensis* was highlighted in this study, suggesting a dilution effect. Indeed, dense cockle populations can filter a high volume of water and thus eliminate parasitic cercariae, with subsequent lower metacercariae infection in cockles (Mouritsen *et al*., 2003; Thieltges and Reise, 2006*b*; Buck and Lutterschmidt, 2017; Magalhães *et al*., 2017; Correia *et al*., 2020*a*). However, the density of cockles only explained 4–8% of metacercariae intensity, implying that other factors, such as host condition, first intermediate host density and environmental parameters modulated the infection of the cockles (Wilson *et al*., 2001; Mouritsen *et al*., 2003; Thieltges and Reise, 2006*b*; Welsh *et al*., 2019).

The phenology's pattern of infection was similar during the first year in both *H. interrupta* and *H. quissetensis*. The infection occurred during the warmer season, as already reported for *H. quissetensis* (Prévot, 1974; Desclaux *et al*., 2004), but also for closely related species such as *Himasthla littorinae* (Nikolaev *et al*., 2020), *H. elongata* (Nikolaev *et al*., 2021), *C. arguinae* (Desclaux *et al*., 2006) and other trematode families such as gymnophallids (Gam *et al*., 2009*b*), renicolids (Thieltges and Rick, 2006) or microphallids (Meißner, 2001). Temperature of water is an important trigger to stimulate infection by cercariae (Lo and Lee, 1996; Mouritsen and Jensen, 1997; Mouritsen, 2002; Koprivnikar *et al*., 2014). In particular, *in situ* experiments showed that the thermal window for cockle infection by *H. quissetensis* was between 15 and 23°C, with maximum infection being at 19–20°C (de Montaudouin *et al*., 2016). During the first winter, the infection intensity per cockle decreased significantly, by 44 and 38% for *H. interrupta* and *H. quissetensis*, respectively. The decrease in the mean parasite intensity could be due to immigration of the less heavily infected cockles or emigration of highly parasitized cockles. This hypothesis seems irrelevant regarding the low locomotive capacity of adult cockles (Richardson *et al*., 1993). It could also be explained by the death of metacercariae in cockles. There are very few studies exploring the dynamics of parasite infrapopulations (i.e. populations at the scale of a host individual). Mortality of parasites was observed in cockles for the non-encysted metacercariae of *G. minutus* (de Montaudouin *et al*., 2012*b*). In this case, the authors had transplanted cockles, and the new site could have been deleterious to parasites, but in other cases *G. minutus* can suffer from hyperparasitism (Fermer *et al*., 2010). However, in the case of *Himasthla* spp. and their encysted metacercariae, empty cysts that suggest parasite death have been observed and registered at a very low intensity (Desclaux *et al*., 2004), leading to the exclusion of this conjecture as well. Finally, the death of the most heavily infected cockles could explain the reduction of *Himasthla* metacercariae intensity in winter. This third hypothesis is the most likely, and has been mentioned in several studies concerning trematodes in their second intermediate hosts (Kennedy, 1984; Desclaux *et al*., 2004, 2006; Gam *et al*., 2009*b*) or first intermediate hosts (Bowers, 1969; Schmidt and Fried, 1997; Rantanen *et al*., 1998; Watters, 1998). During the second summer, the infection pattern was less obvious. It is noteworthy that during summer infections, a stable parasite intensity in cockles can result from a balance between parasite infection and parasite-dependent mortality processes.

### Parasite co-occurrence

The trematode species richness presented in this study was similar to what has been reported in similar ecosystems along the northeast Atlantic coast (Krakau *et al*., 2006; Thieltges *et al*., 2006; Gam *et al*., 2009*b*; Magalhães *et al*., 2018*a*; Correia *et al*., 2020*b*). Negative interactions among parasites within their host have been poorly documented, and in particular few studies have investigated the effect of invasive sporocyst stages on the global diversity of trematodes. Neither *M. parvus* nor *G. choledochus* sporocysts influence the prevalence or abundance of other trematode species (Magalhães *et al*., 2020*a*), contrary to those observed concerning *B. minimus* whose presence is linked to a higher abundance of other trematode species (Magalhães *et al*., 2015). Magalhães *et al*. (2015) suggested that *B. minimus* infection could impair cockle resistance to metacercariae infection, or that high metacercariae infection could facilitate *B. minimus* infestation. However, a second hypothesis is that all parasites co-occur independently of one another, and infect cockles because all conditions are favourable to infection by all parasite species (environmental factors, cockle fitness, other host presence). In the present study, the fact that *H. interrupta* (or *H. quissetensis*) occurrence is associated with higher trematode species richness and abundance, with similar community structure, favours the second hypothesis. Indeed, the relatively low metacercariae intensity values observed, combined with the occupation of specific organs by most trematode species (de Montaudouin *et al*., 2009), are weak arguments supporting an interspecific metacercarial competition, as observed by Thieltges and Reise (2006*b*) and Lassalle *et al*. (2007). In addition, an interspecific competition between *Himasthla* species was not expected, as their metacercariae do not grow inside their second intermediate host (de Montaudouin *et al*., 2005).

### Molecular identity

For *H. elongata*, *H. quissetensis* and *H. interrupta*, the metacercariae molecular identification was performed using 18S and COI sequences. Concerning *H. elongata*, all analysed sequences matched each other, and also sequences that were isolated from *L. littorea* cercariae. These results validate the molecular identification of *H. elongata* since *L. littorea* is the first intermediate host of only this *Himasthla* species. For *H. quissetensis* and *H. interrupta*, the sequences matched each other, and thus provided a good molecular identification. All sequenced *H. quissetensis* came from samples extracted from the cockles' foot, while samples corresponding to *H. interrupta* were extracted from the mantle. However, a mismatch occurred for *H. continua*, with no match among the analysed sequences (high variability). We cannot rule out that this high variability of the obtained sequences was associated with the presence of some larvae of another *Himasthla* species, e.g. *Himasthla leptosoma*. The larvae of these two species are hardly distinguishable by microscopic methods, being very similar in the size of their cysts as well as in the number of spines on the collar (Galaktionov *et al*., 2021). Finally, our results confirm the identity of three species of *Himasthla* metacercariae, which can be difficult to distinguish under a stereomicroscope based on morphological identification.

## Conclusion

Trematodes of the *Himasthla* genus are very common parasites of cockles. Their effect on the cockle individuals or populations is usually reported as low. From an evolution point of view, the metacercarial stage is an opportunity to accumulate diverse parasite genotypes in order to contribute to the genetic diversity of trematode populations (Leung *et al*., 2009). Thus, the main objective of the parasite in this parasitic stage would not be to consume the host energy, as occurs in the first and final hosts. However, a literature review and analysis of a 20-year database revealed that some *Himasthla*-dependent negative effects occur when the metacercariae infection reaches high levels. Considering that this infection is often related to temperature, this parasite dynamics should be monitored according to different climate change scenarios. While morphological identification is particularly difficult concerning *Himasthla* genus, new molecular sequences provided in this study may be helpful for an accurate identification of some species, although uncertainties still remain concerning *H. continua*.

## Data Availability

The data presented in this study are available on request from the corresponding author.
